# Assessment of childhood disease patterns in outpatient settings: a retrospective study from 2016 to 2020

**DOI:** 10.3389/fped.2026.1737317

**Published:** 2026-08-18

**Authors:** Lei Ding, Wencai Ma, Jie Yu, Xiaodan Li, Yulin Sun, Fei Li

**Affiliations:** 1 Department of Pediatrics, the Fifth People’s Hospital of Wujiang District, Suzhou City, Jiangsu Province, China; 2 Tongji University School of Medicine, Shanghai, China; 3 Department of Pediatrics, China-Japan Friendship Hospital, Beijing, China; 4 Department of Pediatrics, the First Medical Center of Chinese PLA General Hospital, Beijing, China; 5 Beijing Children’s Hospital, Capital Medical University, National Center for Children’s Health, Beijing, China

**Keywords:** children, COVID-19, disease distribution, outpatient visit, respiratory diseases

## Abstract

**Background and aim:**

The Coronavirus Disease 2019 (COVID-19) pandemic has significantly impacted pediatric health. Although numerous studies in China have reviewed childhood diseases, specific patterns in certain regions still require more detailed analysis. This was a single-center retrospective study conducted at the Fifth People's Hospital of Wujiang District, Suzhou, Jiangsu, China. It aims to compare the characteristics of pediatric outpatient visits from 2016 to 2019 (pre-pandemic) and 2020 (pandemic), providing insights into how childhood disease patterns have evolved during and after the pandemic.

**Methods:**

We conducted a retrospective analysis to collect routine administrative data on children from outpatient departments

**Results:**

A total of 236,977 visits in outpatient children were recorded, with the highest proportion of visits occurring in 2017 (24.5%) and the lowest in 2020 (11.3%). Changes in visit numbers were mainly associated with fluctuations in respiratory diseases (59.1%). The proportions of respiratory and infectious diseases varied across different years, with the highest proportions observed in 2019 (25.5% and 59.4%, respectively). There was a consistent higher prevalence of male children compared to their female counterparts in terms of demographic characteristics, with a ratio ranging from 1.2 to 1.3. The largest proportion of outpatient visits was observed among preschool-aged children (32.5%), followed by toddlers (26.0%) and school-age children (20.2%). Notably, the fourth quarter consistently had the highest number of visits (30.5%), while the third quarter exhibited the lowest visit numbers (20.0%). Fever (68.6%) was the most frequently reported symptom in outpatient visits with abnormal symptoms and signs.

**Conclusions:**

This study highlights the impact of the COVID-19 pandemic on outpatient visits among children in Wujiang District, Suhzou, revealing a downward trend in 2020 compared to previous years. Notably, respiratory diseases remained the most common reason for outpatient visits, with distinct demographic patterns observed. These findings may help inform local healthcare planning and resource allocation in similar settings.

## Introduction

From December 2019 onwards, there has been a global outbreak of coronavirus disease 2019 (COVID-19) (1, 2). In China, nationwide non-pharmaceutical measures were put in place and were eased in April 2020 after successfully controlling the spread of COVID-19 for the moment (3). Multiple studies have shown that these substantial public health interventions have been associated with changes in human behavior and have also affected outpatient and hospitalization patterns for other illnesses, particularly respiratory diseases, because their transmission routes are similar to those of severe acute respiratory syndrome coronavirus 2 (SARS-CoV-2) (4–7). Acute respiratory infections (ARI), particularly viral lower respiratory tract infections, are the leading causes of illness and death in children under 5 years old (8, 9).

Children play a crucial role in achieving herd immunity against respiratory and gastrointestinal viruses, thereby serving as a reflection of the prevalence of these viruses within the community (10). Moreover, sudden changes in personal hygiene practices and stringent epidemic prevention and control measures can profoundly influence the customary occurrence of respiratory and gastrointestinal diseases, along with other ailments, within the population (11, 12). This phenomenon is supported by comparative analyses of disease prevalence characteristics in the pre- and post-COVID-19 periods (4, 13, 14). Despite the high number of children seeking medical treatment for various diseases, there is still a lack of comprehensive data on the specific characteristics of these diseases in China.

Although several studies have reported the impact of the COVID-19 pandemic on pediatric healthcare utilization in China, most were conducted in tertiary pediatric centers or focused on specific infectious diseases. Data from regional general hospitals and district-level outpatient settings remain relatively limited. In addition, few studies have provided continuous comparisons of pediatric outpatient disease patterns before and during the pandemic across multiple years. Therefore, understanding local disease distribution and healthcare utilization patterns may provide important evidence for regional pediatric healthcare planning and resource allocation. Therefore, this study aimed to describe the characteristics and distribution of disease categories among pediatric outpatients in our single tertiary hospital in Suzhou, Jiangsu, China.

## Methods

### Study design and participants

We designed this study to collect routine administrative data from the Fifth People's Hospital of Wujiang District, Suzhou, Jiangsu, China. Data on visits by children aged ≤18 years who sought medical treatment at the hospital, were collected from the of the outpatient department's hospital information system between January 1, 2016, and December 31, 2020. Relevant information was extracted based on ICD-10 (International Statistical Classification of Diseases and Related Health Problems, 10th Revision) codes (https://icd.who.int/browse10/2019/en), such as respiratory diseases (J00-J99), diseases of the digestive system (K00-K93), among others. ICD-10 diagnostic codes were assigned by attending clinicians during routine clinical practice and recorded in the hospital information system. Disease categories used in this study were subsequently grouped by the researchers according to standardized ICD-10 classifications. Specifically, each outpatient visit was assigned based on the main diagnosis. Outpatient diagnoses and encounter types were classified into 17 categories, including respiratory system diseases (e.g., pneumonia), abnormal symptoms and signs (e.g., fever), ear nose and throat diseases (e.g., otitis media), digestive system diseases (e.g., gastritis), infectious diseases (e.g., influenza), skin and allergic diseases (e.g., urticaria), neonatal diseases (e.g., neonatal jaundice), parasitic diseases (e.g., intestinal parasitic infection), endocrine system diseases (e.g., diabetes mellitus), health examination (physical examination/health check-up visit), urinary system diseases (e.g., acute glomerulonephritis), blood system diseases (e.g., leukemia), nervous system diseases (e.g., epilepsy), circulatory system diseases (e.g., congenital heart disease), diseases caused by various physical and chemical factors (including poisoning, injuries, insect bites, and burns), rheumatic immune diseases (e.g., systemic lupus erythematosus), and other. Abnormal symptoms and signs were used to group outpatient encounter entries primarily characterized by symptoms or abnormal clinical signs and abnormal clinical findings. This category can be identified as symptoms, signs, or abnormal presentations (e.g., fever, abdominal pain, vomiting). COVID-19 cases with ICD-10 codes U07.1 (COVID-19, virus identified) and U07.2 (COVID-19, virus not identified) were classified under the respiratory system disease. Diagnoses that can be clearly categorized into other disease systems were not assigned to this category. Demographic information, such as age, sex, date of birth, date of outpatient clinic visit, and diagnosis, were included. Repeated registrations from the same child on the same day were considered a single outpatient visit, whereas visits occurring more than one week apart were retained for analysis. According to different ages, children were divided into six groups, including neonate (≤28 days), infant (>28 days to ≤1 year old, neonate was excluded), toddler (>1 to ≤3 years old), preschooler (>3 to ≤6 years old), school-age children (>6 to ≤12 years old), and adolescents (>12 to <18 years old). This study defines a year as comprising four quarters: the first quarter spans from January to March, the second quarter from April to June, the third quarter from July to September, and the fourth quarter from October to December.

### Statistical analysis

IBM SPSS Statistics 23.0 software (SPSS Inc., USA) was used for data analysis. Categorical variables were expressed as number (%) and compared between groups by Chi-square test or Fisher's exact test, when appropriate.

## Results

### Overview of visits from patients

**Figure 1:**
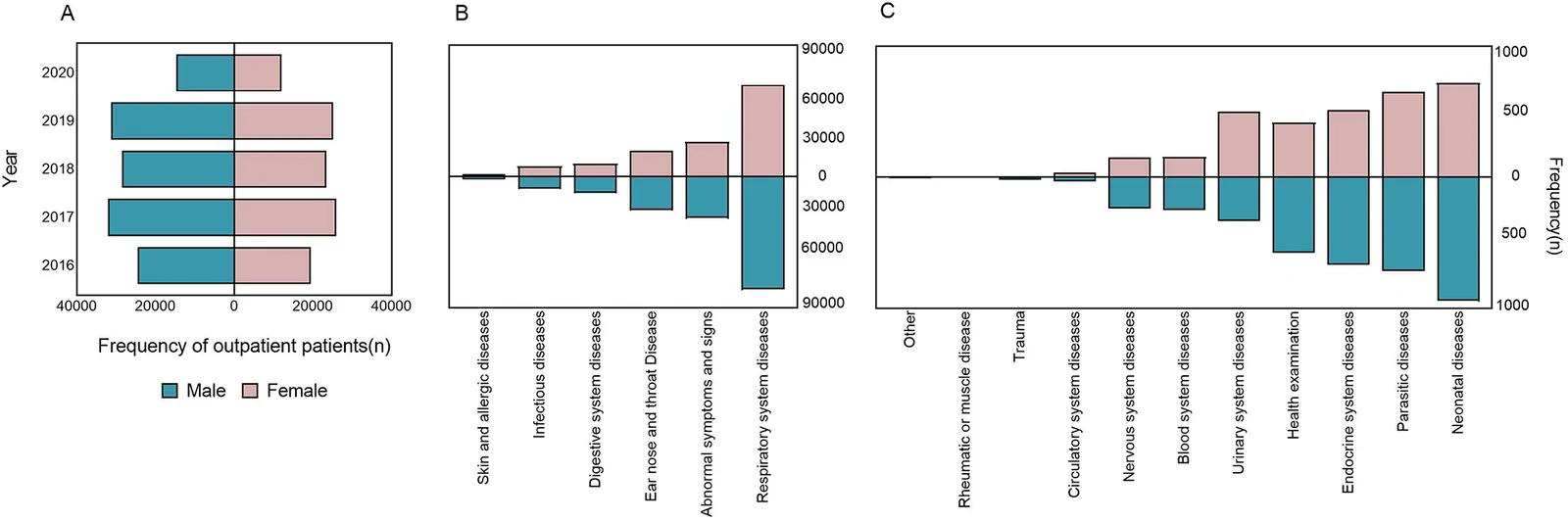
Gender composition of pediatric patients in outpatient departments in Suzhou 2016-2020. **(A)** Annual distribution; **(B,C)** Distribution of detailed disease system classifications (shown in two panels for clarity).

From 2016 to 2020, 236,977 outpatient clinic visits were recorded in the Hospital. All visits were included in the analysis. The total number of visits fluctuated, reaching the highest in 2017 with 57,989 visits (24.5%) and the lowest in 2020 with 26,773 visits (11.3%). Respiratory system diseases accounted for 59.1% (140,082 cases) of all visits, while abnormal symptoms and signs accounted for 22.0% (52,140 cases), ear nose and throat disease for 17.10% (40,529 cases), digestive system diseases for 8.4% (19,830 cases), and infectious diseases for 6.4% (15,248 cases) (Table 1, Figure 1).

**Table 1:** Overview of visits from patients in suzhou, Jiangsu, China, 2016 to 2020. (n, %).

Characteristic	2016 (*n* = 43,994)	2017 (*n* = 57,989)	2018 (*n* = 51,896)	2019 (*n* = 56,325)	2020 (*n* = 26,773)
Gender					
male	24,579 (55.9)	32,080 (55.3)	28,521 (55.0)	31,248 (55.5)	14,696 (54.9)
female	19,415 (44.1)	25,909 (44.7)	23,375 (45.0)	25,077 (44.5)	12,077 (45.1)
Age					
neonate	3,098 (7.0)	3,157 (5.4)	2,384 (4.6)	2,241 (3.0)	2,632 (9.8)
infant	7,298 (16.6)	9,986 (17.2)	7,086 (13.7)	6,608 (11.7)	2,840 (10.6)
toddler	12,098 (27.5)	14,986 (25.8)	13,572 (26.2)	14,804 (26.3)	6,145 (22.0)
preschooler	13,930 (31.7)	18,213 (31.4)	18,166 (35.0)	18,856 (33.5)	7,780 (29.1)
school-age children	7,215 (16.4)	10,934 (18.9)	9,947 (19.2)	13,038 (23.1)	6,720 (25.1)
adolescence	355 (0.8)	713 (1.2)	741 (1.4)	778 (1.4)	656 (2.5)
Quarter					
Q1	10,395 (23.6)	14,260 (24.6)	14,208 (27.4)	14,601 (25.9)	6,172 (23.1)
Q2	10,324 (23.5)	15,246 (26.3)	13,901 (26.8)	14,310 (25.4)	4,030 (15.1)
Q3	8,728 (19.8)	14,260 (24.6)	10,295 (19.8)	10,810 (19.2)	6,557 (24.5)
Q4	14,547 (33.1)	17,595(30.3)	13,492(26.0)	16,604(29.5)	10,014(37.4)

The terms Q1, Q2, Q3, and Q4 correspond to the first quarter (January-March), second quarter (April-June), third quarter (July-September), and fourth quarter (October-December), respectively.

### Demographic characteristics of patients

Of these visits, 131,124 (55.3%) were made by male children and 105,853 (44.7%) were made by female children. During the five-year period, male children consistently outnumbered female children during the five-year period, with a fluctuating ratio of male to female children ranging from 1.2 to 1.3. Similarly, this male-to-female ratio remained within 1.2-1.3 across different age groups. Except for urinary and reproductive system diseases, male children had higher numbers and proportions of visits to the outpatient department compared to female children, with a ratio ranging from 1.1 to 1.6 (Table 1, Figure 1).

**Figure 2:**
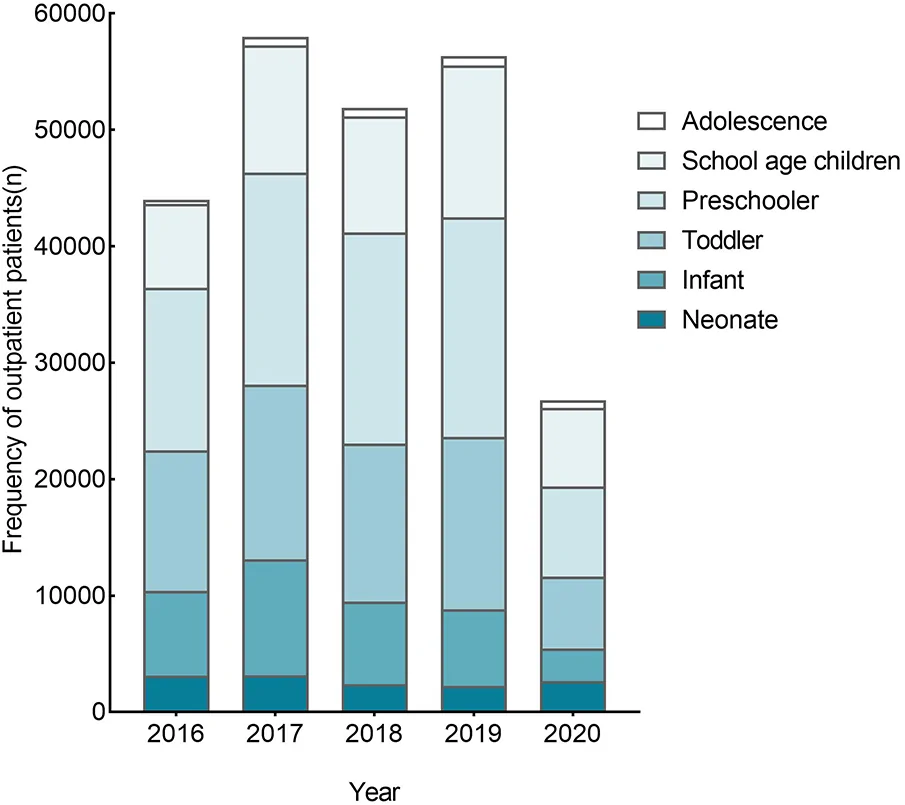
Age composition of outpatient patients in different years. Six groups: neonate (≤28 days), infant (>28 days to ≤1 years old, neonate was excluded), toddler (>1 to ≤3 years old), preschooler (>3 to ≤6 years old), school-age children (>6 to ≤12 years old), adolescence (>12 to <18 years old).

The breakdown by age groups in descending order is as follows: preschoolers accounted for the highest number of visits with 76,945 (32.5%), followed by toddlers with 61,605 visits (26.0%), school-age children with 47,854 visits (20.2%), infants with 33,818 visits (14.3%), neonates with 13,512 visits (5.7%), and adolescents accounted for the lowest number of visits with 3,243 (1.4%). Notably, the proportion of preschoolers consistently remains highest, ranging from 29.1% to 35.0% across different years (Figure 2).

For the distribution by quarters of five years, the 4th quarter recorded the highest number of visits with 72,252 (30.5%), followed by the 1st quarter with 59,636 visits (25.2%), the 2nd quarter with 57,811 visits (24.4%), and the 3rd quarter had the lowest number of visits with 47,278 (20.0%) (Table 1). Over a five-year period, the fourth quarter consistently exhibits the highest proportion of visits (29.5%–37.4%). Except for 2020, the third quarter consistently records the lowest number of outpatient visits (19.2%–24.6%) (Table 1).

### Distribution characteristics of different diseases

**Figure 3:**
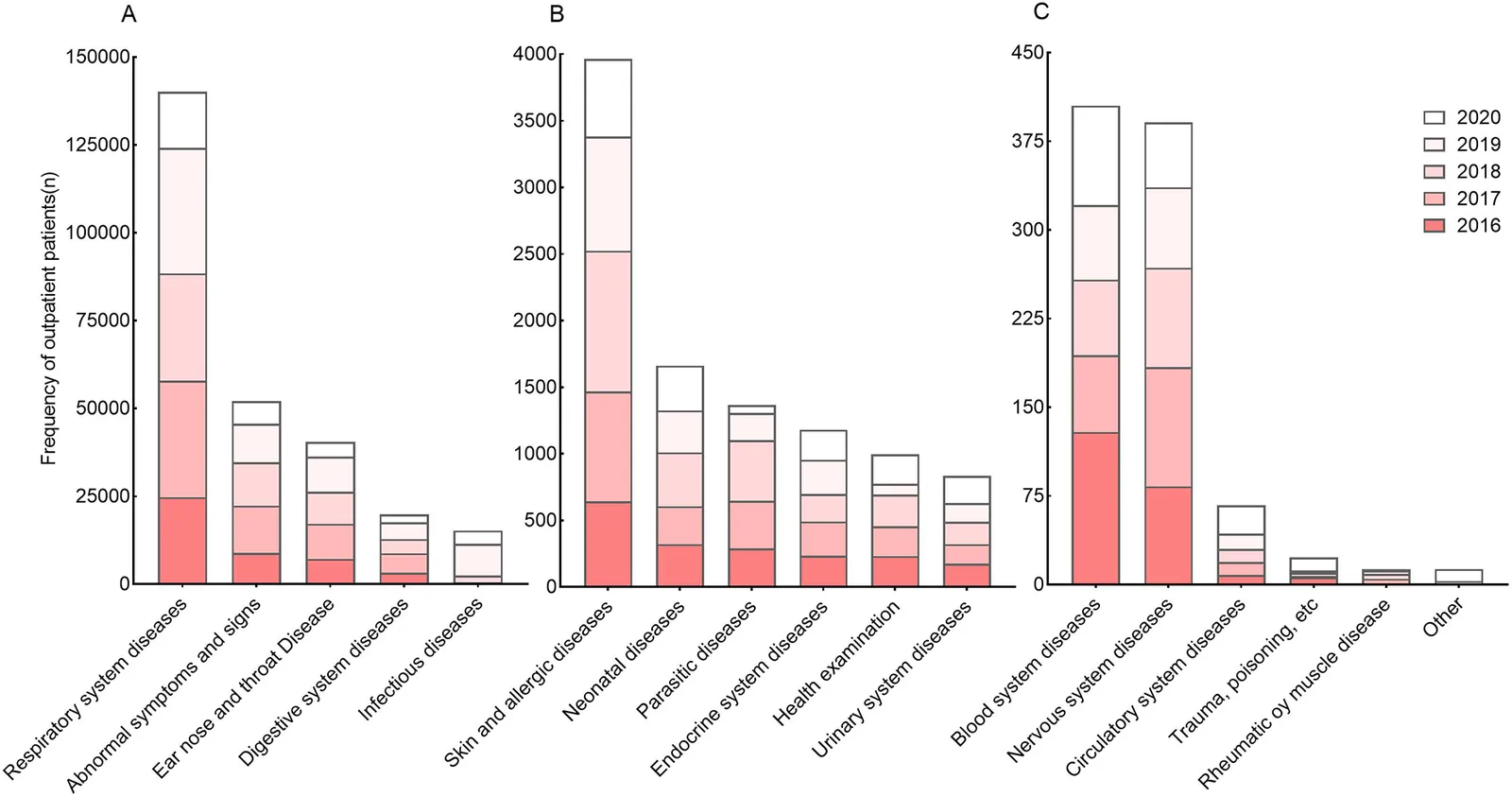
Different disease distributions of outpatient patients in different years. **(A)** The top five disease categories by frequency; **(B)** The six mid-ranked disease categories; **(C)** The six lowest-ranked disease categories.

The diagnosed diseases of pediatric patients visiting the hospital have been classified into 17 categories (Figure 3). The categories are ranked in descending order based on patient numbers as follows: respiratory system diseases (59.1%, 140,082 cases), abnormal symptoms and signs (22.0%, 52,140 cases), ear nose and throat diseases (17.1%, 40,529 cases), digestive system diseases (8.4%, 19,830 cases), infectious diseases (6.4%, 15,248 cases), and skin and allergic diseases (1.7%, 3,965 cases). Among the other 11 categories, it accounts for less than 1%.

The frequency distribution of different disease classifications in different years was depicted in Figure 3. Among them, respiratory system diseases (35,697/140,082, 25.5%) and infectious diseases (9,050/15,248, 59.4%) recorded the highest proportions in 2019. In contrast, the year 2017 witnessed the highest proportions of abnormal symptoms and signs, ear nose and throat disease, and digestive system diseases, which were 25.7% (13,414/52,140), 24.6% (9,979/40,529), and 28.1% (5,568/19,830), respectively. Skin and allergic diseases peaked in 2018 with a proportion of 26.6% (1,056/3,965).

**Figure 4:**
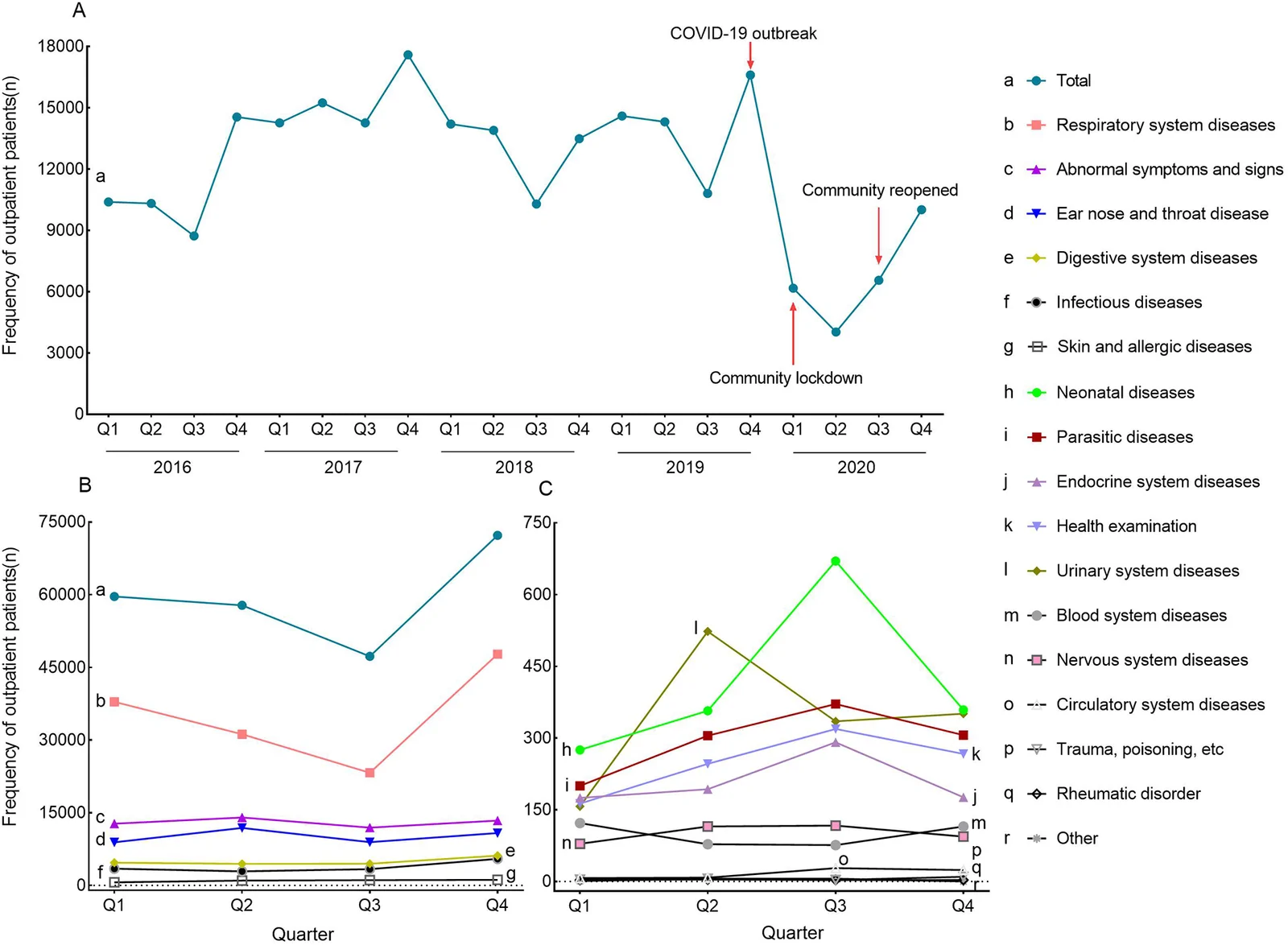
Different disease distributions of outpatient patients in different quarters from 2016 to 2020. **(A)** Quarterly distribution across different years; **(B,C)** Overall quarterly distribution across the study period (shown in two panels for clarity). Four quarters: the first quarter spans from January to March, the second quarter from April to June, the third quarter from July to September, and the fourth quarter from October to December.

Regarding the annual distribution, fluctuations were observed in the number of outpatient department visits from 2016 to 2020 as evidenced in Figure 4, ranging from 4,030 visits in 2020 to 17,595 visits in 2017, with the total number of patients in 2020 being less than in the fourth quarter of 2017 (Figure 4A). Throughout the years 2016–2019, the number of outpatient department visits consistently reached its lowest point during the third quarter (July-September). Towards the end of 2019, the outbreak of COVID-19 caused a sudden upsurge in outpatient volume, prompting the implementation of control measures by the Chinese government. Consequently, the number in 2020 reached their lowest level in nearly five years. As nationwide restrictions gradually eased, the number of outpatient visits experienced a progressive recovery. The quarterly distribution chart of outpatient department visits underscored that fluctuations in visit numbers were predominantly driven by changes in respiratory diseases (Figures 4B,C).

**Figure 5:**
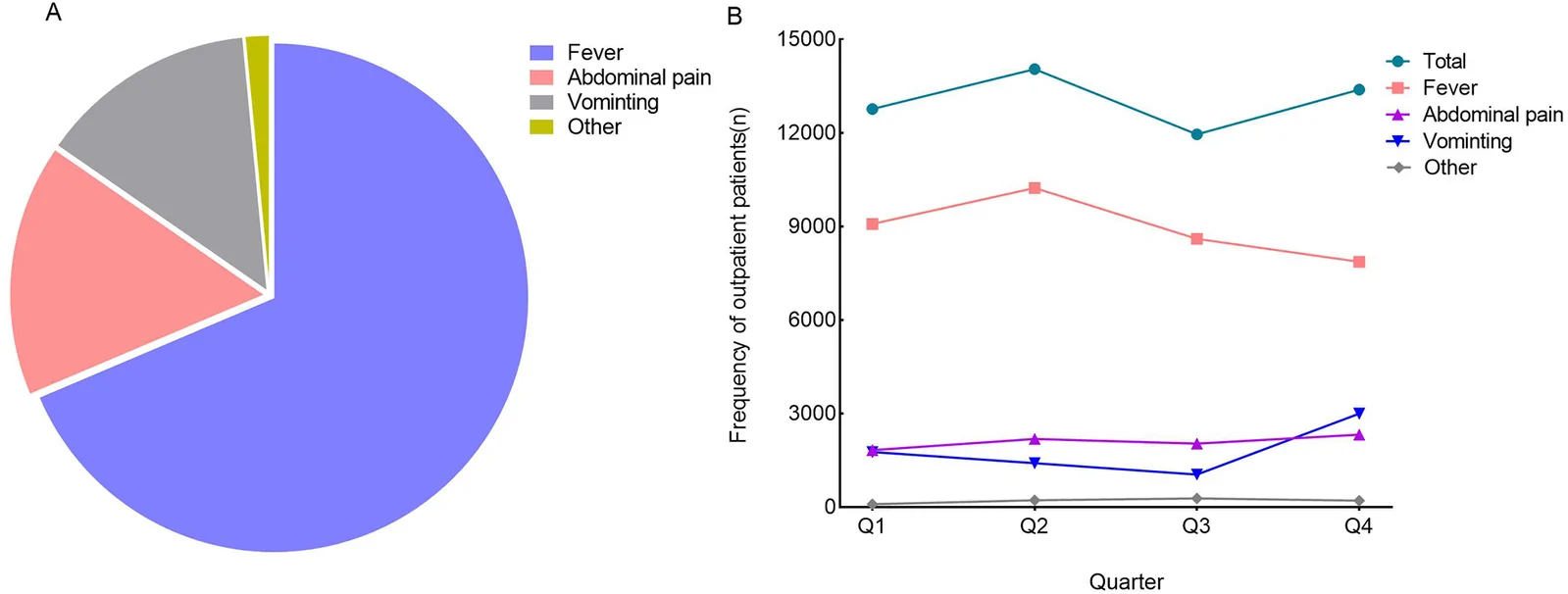
The composition for outpatient patients seeking medical attention due to abnormal symptoms and signs. **(A)** Overall proportions of visits categorized by symptoms (fever, abdominal pain, vomiting, and other). **(B)** Quarterly trends (Q1-Q4) in visit frequencies for each symptom category and for the total number of outpatient visits. The first quarter (Q1) spans from January to March, the second quarter (Q2) from April to June, the third quarter (Q3) from July to September, and the fourth quarter (Q4) from October to December.

A subset of patients who seek outpatient care primarily present with symptoms, and we classify them as “abnormal symptoms and signs” (22.0%, 52,140 cases). This category encompasses symptoms such as fever, abdominal pain, vomiting, and others, accounting for 68.6% (35,774 cases), 16.0% (8,361 cases), 13.8% (7,209 cases), and 1.5% (796 cases), respectively (Figure 5A). The male-to-female ratio ranges from 1.2 to 1.4. The second quarter (April to June) exhibited the highest patient volume across the four quarters of the year, primarily due to fluctuations in fever cases (Figure 5B).

## Discussion

The retrospective analysis of outpatient clinic visits from 2016 to 2020 reveals significant trends and patterns in pediatric healthcare utilization. During this five-year period, a total of 236,977 visits were recorded, with the peak occurring in 2017 and a decline in 2020. Respiratory system diseases were the leading cause for these visits. The demographic analysis showed a consistent male children predominance among pediatric patients, with preschoolers representing the largest age group seeking outpatient care. Additionally, the distribution of visits across different quarters demonstrates seasonal patterns, particularly in respiratory and infectious diseases.

Respiratory diseases have been the leading reason for medical visits in children in recent decades (15–18). This study found that from 2016 to 2020, patients with respiratory diseases account for a large proportion of pediatric outpatient visits. This may be related to the incomplete development of the children's immune systems and certain physiological structures (19, 20). Li et al. and Zhao et al. reported that in the aftermath of the COVID-19 pandemic and subsequent easing of containment measures, multiple respiratory pathogens (including RSV, ADV, and Influenza A virus) re-emerged sequentially. Their studies revealed that infection rates exceeded those observed during the pandemic period, with a notable shift in outbreak temporality, manifesting earlier than historical seasonal patterns (4, 13). Previous studies demonstrated that respiratory diseases were the most prevalent diseases, in low- and middle-income countries (9, 10). Numerous studies have consistently demonstrated that respiratory diseases pose a significant health burden on children, exceeding that of other diseases (21–23). Therefore, adequate allocation of pediatric respiratory care resources, including staffing and seasonal preparedness, may be particularly important in district-level hospitals. This study found that respiratory illnesses were the most common (59.1%), followed by unclassified illnesses (primarily fever-related) (22.0%) and illnesses from the eyes, ears, nose, and throat (17.0%). Most studies have found that infectious diseases are the most common cause of Fever of Unknown Origin (FUO) in children visiting hospitals, followed by rheumatic diseases and tumors (24, 25). In our study, 35,774 patients (68.6%) with unclassified diseases during outpatient visits presented with fever, suggesting a significant portion may have infectious diseases. However, follow-up is essential to avoid missing diagnoses of tumors and other conditions, thereby ensuring effective treatment.

A disparity in patient gender was observed, with male children outnumbering female children by approximately 1.2 times across different years, age groups and disease categories. This disparity can be attributed to various factors. For example, boys are more likely to engage in risky behaviors, which contributes to the higher incidence of health issues in this demographic. Moreover, this preponderance appears to be a global phenomenon, as it has been widely reported in various studies (26, 27). Research indicates that the incidence of urinary tract infections (UTIs) varies by age and gender (28). In the first year of life, boys experience a higher incidence of UTIs compared to girls (3.7% vs. 2%). However, during the preadolescent years, girls have a higher incidence of UTIs compared to boys (approximately 3% vs. 1%) (28, 29). Our study shows comparable trends. Specifically, we observed a higher proportion of female children seeking treatment for urinary system diseases. This difference may be related to the age distribution of our sample: neonates accounted for 5.7%, while preschool children accounted for 32.5%.

The number of children seeking medical attention at outpatient departments exhibited a relatively stable pattern between 2016 and 2019; however, a shift occurred in 2020 in regional pediatric outpatient settings. The reduction in pediatric outpatient visits observed in 2020 was likely multifactorial, potentially reflecting the combined effects of public health interventions, altered healthcare-seeking behavior, and decreased transmission of common infectious diseases during the COVID-19 pandemic, similar to previous studies (30–32). Recent studies have further suggested that socioeconomic factors, including parental education level and household income, may influence children's healthcare utilization and healthcare-seeking behavior during the COVID-19 pandemic. Families from different socioeconomic backgrounds may differ in health awareness, healthcare accessibility, and willingness to seek medical care during public health emergencies, which may also partly contribute to changes in pediatric outpatient attendance (33, 34). However, because socioeconomic data were unavailable in the present study, further studies are needed to clarify their impact on pediatric outpatient utilization in regional healthcare settings. As reported by Paula et al., in their study, the peak of ALRI-related visits occurred during the cold months before 2020, disappeared in 2020, re-emerged with a delay in 2021, and returned to its seasonal pattern in 2022 (35). This provides a useful context for understanding how pandemic-related changes may influence healthcare-seeking patterns for respiratory infectious diseases in children. These findings may inform public health planning and outpatient service preparedness during similar periods. It has been reported that implementing long-term community measures to mitigate the spread of COVID-19 has resulted in a significant reduction in the transmission of respiratory and gastrointestinal viruses (36–38). Figure 3 reveals a downward trend in the number of children and adolescents seeking medical care for respiratory diseases, abnormal symptoms and signs (particularly fever), otorhinolaryngology diseases, digestive system diseases, and infectious diseases in 2020, as compared to preceding years, which was similar to previous reports (11, 39, 40). Our study observed that the total number of outpatient visits reached its lowest point in 2020, which coincided with the implemented disease control measures. Additionally, there was a resurgence in the number of patient visits following the reopening of communities, which was also observed in other studies (4, 35, 41). Because detailed data regarding healthcare-seeking behavior, policy implementation intensity, and community-level disease incidence were unavailable, the relative contribution of these factors could not be quantified in the present study. These findings indicate that primary and regional pediatric services are highly sensitive to large-scale public health events.

There were a larger number of children in the preschool period (>1 to ≤3 years old) and the early childhood period (>3 to ≤6 years old), with 61,605 and 76,945 visits respectively, accounting for 58.7% of the total population. Many studies have pointed out that globally, children under the age of 5 years old remain one of the high-risk age groups for various diseases, suggesting that this population may require greater attention in regional pediatric healthcare planning and infectious disease prevention strategies. In this age group, acute lower respiratory infections identified as one of the primary causes for the high incidence and mortality rates in this age group, followed by digestive system diseases (42–44). Several factors may explain this pattern. First, children in this age group are at a crucial stage of immune system development, making them more vulnerable to various infections (19). Second, preschool-aged children commonly attend childcare centers or kindergartens, where they have frequent close contact with peers, thereby increasing the risk of infectious disease transmission (45). Moreover, the vaccination coverage for children in this specific period remains incomplete, increasing their propensity to seek medical care in hospitals when compared to children in differing age cohorts (46, 47).

Compared to other diseases, the high incidence of respiratory diseases in children during medical visits primarily arises from physiological factors, the contagious nature of respiratory disease transmission, increased exposure due to environmental changes, and heightened attention from caregivers prompting medical consultations (48, 49). Together, these factors contribute significantly to the frequency of healthcare visits for respiratory ailments in this population. A significant correlation exists between changes in patient numbers and respiratory diseases, highlighting the impact of these factors on healthcare utilization. Furthermore, Zhang et al. reported that the reduction of airborne transmission plays a crucial role in the control of infectious respiratory diseases (50). Respiratory diseases significantly increase in children during cold and humid weather (51). In this study, the number of visits for children with fevers, otorhinolaryngology diseases, gastrointestinal diseases, and infectious diseases remained stable across different quarters. This finding contrasts with other research that typically shows seasonal changes in disease incidence, especially infectious diseases (52, 53). The discrepancy may be due to our selection of outpatient records, which might not fully capture seasonal trends seen in other types of medical data.

This study has some limitations. Firstly, this study was conducted in a single healthcare setting and focused on outpatient patients, while neglecting the hospitalized pediatric patients during the same period. This may have led to a failure to fully reflect the overall situation of children seeking medical treatment. Secondly, this primarily observational study acknowledges the possibility of diagnostic misclassification, making it difficult to establish causality and only allowing for the identification of potential correlations through observation. Moreover, because this study was based on secondary analysis of a limited set of variables, we could not capture important information such as follow-up outcomes, disease resolution, or treatment trajectories. Third, we only classified the diseases of the patients without deep analysis of the most prevalent respiratory diseases, particularly the etiology, which may hinder the guidance for better treatment.

## Conclusion

The COVID-19 pandemic has had a notable impact on pediatric outpatient visits in children. Despite this, respiratory diseases remain the most common reason for seeking medical attention. In addition, the study found differences in the number of visits across quarters. Overall, these findings can provide valuable insights into the patterns and trends of pediatric outpatient visits in Suzhou, as well as the impact of the pandemic of infectious diseases on the health of children.

## Data Availability

Publicly available datasets were analyzed in this study. This data can be found here: The raw data supporting the conclusions of this article will be made available by the authors, without undue reservation.
